# Genome-wide association analysis identifies a consistent QTL for powdery mildew resistance on chromosome 3A in Nordic and Baltic spring wheat

**DOI:** 10.1007/s00122-023-04529-1

**Published:** 2024-01-19

**Authors:** Min Lin, Bulat Islamov, Andrius Aleliūnas, Rita Armonienė, Andrii Gorash, Egon Meigas, Anne Ingver, Ilmar Tamm, Hannes Kollist, Vija Strazdiņa, Māra Bleidere, Gintaras Brazauskas, Morten Lillemo

**Affiliations:** 1 Department of Plant Sciences, Norwegian University of Life Sciences, Post Box 5003, NO-1432 ÅS, Norway; 2 Centre of Estonian Rural Research and Knowledge, J. Aamisepa 1, Jõgeva Alevik, 48309 Jõgeva Maakond, Estonia; 3 Institute of Agriculture, Lithuanian Research Centre for Agriculture and Forestry, 58344 Akademija, Lithuania; 4 Institute of Bioengineering, University of Tartu, Nooruse 1, 50411 Tartu, Estonia; 5 Institute of Agricultural Resources and Economics, Zinatnes Iela 2, Cesis County, 4126 Latvia

## Abstract

**Key message:**

*QPm.NOBAL-3A* is an important QTL providing robust adult plant powdery mildew resistance in Nordic and Baltic spring wheat, aiding sustainable crop protection and breeding.

**Abstract:**

Powdery mildew, caused by the biotrophic fungal pathogen *Blumeria graminis* f. sp. *tritici*, poses a significant threat to bread wheat (*Triticum aestivum* L.), one of the world’s most crucial cereal crops. Enhancing cultivar resistance against this devastating disease requires a comprehensive understanding of the genetic basis of powdery mildew resistance. In this study, we performed a genome-wide association study (GWAS) using extensive field trial data from multiple environments across Estonia, Latvia, Lithuania, and Norway. The study involved a diverse panel of recent wheat cultivars and breeding lines sourced from the Baltic region and Norway. We identified a major quantitative trait locus (QTL) on chromosome 3A, designated as *QPm.NOBAL-3A*, which consistently conferred high resistance to powdery mildew across various environments and countries. Furthermore, the consistency of the QTL haplotype effect was validated using an independent Norwegian spring wheat panel. Subsequent greenhouse seedling inoculations with 15 representative powdery mildew isolates on a subset of the GWAS panel indicated that this QTL provides adult plant resistance and is likely of race non-specific nature. Moreover, we developed and validated KASP markers for *QPm.NOBAL-3A* tailored for use in breeding. These findings provide a critical foundation for marker-assisted selection in breeding programs aimed at pyramiding resistance QTL/genes to achieve durable and broad-spectrum resistance against powdery mildew.

**Supplementary Information:**

The online version contains supplementary material available at 10.1007/s00122-023-04529-1.

## Introduction

Bread wheat, *Triticum aestivum* L., is the most grown cereal crop worldwide (FAO 2021). However, wheat production faces significant challenges due to various fungal diseases, including powdery mildew caused by the fungal pathogen *Blumeria graminis* f. sp. *tritici,* which is common in the Nordic and Baltic region. As a biotrophic pathogen, it infects the epidermal cells of wheat leaves and requires living host cells to survive and complete its life cycle. Race-specific resistance is common against powdery mildew (Pm). This type of resistance is governed by major resistance genes, which can confer specific protection against the pathogen by recognition of specific effectors produced by particular pathogen races, following the classical gene-for-gene model (Flor 1971).

Deploying resistant cultivars is an environmentally sustainable approach to manage powdery mildew disease, and the characterization of *Pm* genes has greatly contributed to our understanding of the mechanisms underlying race-specific resistance. Such approaches lead to improved management strategies in wheat breeding programs. So far a dozen of *Pm* genes have been cloned, such as *Pm2* (Sanchez-Martin et al. 2016), *Pm3* (Yahiaoui et al. 2004), *Pm4* (Sanchez-Martin et al. 2021), *Pm5* (Xie et al. 2020), *Pm8* (Hurni et al. 2013), *Pm17* (Singh et al. 2018), *Pm21* (He et al. 2018), *Pm24* (Lu et al. 2020), *Pm38* (Krattinger et al. 2009), *Pm41* (Li et al. 2020), *Pm46* (Moore et al. 2015), and *Pm60* (Zou et al. 2018), reviewed by Hinterberger et al. (2022).

*Pm3* was the first extensively studied *Pm* gene against wheat powdery mildew (Yahiaoui et al. 2004), and belongs to a gene family characterized by multiple alleles, each conferring resistance against specific races (Koller et al. 2018). As a typical race-specific resistance gene, *Pm3* is characterized by the presence of nucleotide-binding site (NBS) and leucine-rich repeat (LRR) domains. The majority of well-documented race-specific resistance genes against powdery mildew belong to this gene family, although exceptions exist, including *Pm4*, which encodes a putative chimeric protein comprising multiple C2 domains and transmembrane regions in addition to a serine/threonine kinase (Sanchez-Martin et al. 2021), and *Pm24*, which encodes a tandem kinase protein (TKP) (Lu et al. 2020).

However, the effectiveness of single race-specific resistance genes has typically short durability and can be overcome after only a few years of wide deployment due to the coevolution of the pathogen population, as demonstrated by examples such as *Pm8* and *Pm17* (Cowger et al. 2009; Kunz et al. 2023). Therefore, there is a growing interest in utilization of race non-specific resistance genes, which provide more durable resistance. Numerous studies have been conducted to understand the molecular mechanisms of different types of resistance. When comparing gene expression patterns of both types of resistance via transcriptomic analysis, it was found that race non-specific resistance involves a larger number of genes and transcripts than race-specific resistance. Additionally, adult plant resistance genes are induced more slowly than genes involved in all-stage resistance (Chen et al. 2013; Tal et al., 2018). However, the complete molecular mechanisms underlying the race non-specific powdery mildew resistance remain largely unknown. The nature of all identified *Pm* genes is not known yet, and the distinction between race-specific and race non-specific genes is not always crystal clear. However, expression stage of resistance is one of the main attributes used to categorize resistance as adult plant resistance (race non-specific) or all-stage resistance (race-specific) (Wang et al. 2023). Currently, the molecular structure is only known for two race non-specific resistance genes against wheat powdery mildew, namely *Pm38* and *Pm46*, and these genes confer pleiotropic resistance against multiple fungal diseases. *Pm38*, also known as *Lr34/Yr18/Sr57*, encodes an ABC transporter and has shown resistance against wheat leaf rust, stripe rust, stem rust and powdery mildew (Krattinger et al. 2009). *Pm46*, also known as *Lr67/Yr46/Sr55*, encodes a hexose transporter and confers resistance against all three wheat rust diseases in addition to powdery mildew (Moore et al. 2015).

In order to enhance cultivar resistance against powdery mildew, an effective strategy is pyramiding race-specific resistance genes while also exploring additional race non-specific resistance genes. The objectives of the present study were to map the main genetic loci for powdery mildew field resistance in a panel of recent cultivars and breeding lines from the Baltic countries and Norway through field testing in multiple environments across Estonia, Latvia, Lithuania and Norway. Greenhouse seedling inoculations with multiple representative powdery mildew isolates were used to validate whether the major QTL *QPm.NOBAL-3A* detected in this study conferred adult plant resistance.

## Materials and methods

### Plant materials and genotyping

The NOBALwheat association mapping panel was assembled of 300 spring wheat accessions of which 170 were breeding lines and 130 cultivars with a release year ranging from 1904 to 2022, while the majority of the collection were new lines released after 2010. The accessions were selected as a representative set for the breeding pools of NOBALwheat partner institutions, e.g., 73 accessions from each partner and 10 lines with exotic sources from CIMMYT, China and Australia. For genotyping, a single typical spike of each accession from NOBALwheat collection was collected from a field trial at the Lithuanian Research Centre for Agriculture and Forestry (LAMMC) in 2021. Five seeds from each spike were sown in pots and were grown till the two-leaf stage. The leaf tissue from the resulting five plants was sampled, pooled together into the collection plates, and dried at 60 °C for 12 h. Dried leaf samples were sent to TraitGenetics GmbH, Germany (http://www.traitgenetics.com/en/) for further DNA extraction and genotyping with a 25 K SNP wheat marker array. Genotypic data was filtered to exclude failed or monomorphic markers. In addition, genetic markers with more than 20% of missing data were also removed. Heterozygous genotypes were treated as missing. After filtering out markers with allele frequency less than 5%, 18562 markers were left for further genetic analysis. For validating the GWAS results, the NMBU spring wheat panel, consisting of 300 spring wheat lines, was genotyped by the same wheat 25 K SNP chip from TraitGenetics, as described by Lin et al. (2022).

### Field trials

Ten field trials for NOBALwheat panel addressing powdery mildew adult plant resistance were carried out in the 2021 and 2022 field seasons in three Baltic states (Estonia, Latvia, and Lithuania) and Norway (Fig. S1). The powdery mildew disease trials in Estonia were carried out as hillplots in two replicates by natural infection. Twenty seeds per hillplot were sown manually. Later than optimal (May 12 in 2021 and May 19 in 2022) sowing time was used to promote disease infection. Trials were carried out at Centre of Estonian Rural Research and Knowledge, Jõgeva (58°46′ N, 26°24′ E), Estonia (EE_2021, EE_2022). Four disease evaluations were done in both years on the following dates: July 02, 09, 16 and 22 in 2021 and July 14, 21, 29 and August 05 in 2022. Disease trials in Latvia were arranged in 1 m^2^ plots with randomized complete-block design of two replicates by natural infection. The trials were conducted in year 2021, 2022 at Stende Research Centre (57°11′ N, 22°32′ E) of Institute of Agricultural Resources and Economics, Latvia (LV_2021, LV_2022). The powdery mildew disease trials in Norway were carried out as hillplots with alpha lattice design of two replicates by natural infection, in the years 2021 and 2022 at Vollebekk (59°39′ N, 10°45′ E) in Ås, Viken, Norway (NO_2021_Vol, NO_2022_Vol), and Staur (60°44′ N, 11°06′ E), Innlandet, Norway (NO_2021_St, NO_2022_St). In Norway, disease scoring was carried out when the susceptible check line ‘Avocet YrA’ reached 70–100%. Disease trials in Estonia, Latvia and Norway were based on visual scorings of the disease severity on the percentage scale, where 0 represents no infection and 100% represents heavy infection. In Lithuania, disease trials were carried out in 1.5 square meter plots in alpha lattice design with two replicates under natural infection in Dotnuva (55°24′ N, 23°52′ E) in year 2021 and 2022 (LT_2021, LT_2022). Visual scoring of powdery mildew was performed three times in 2021 (June 18th, 28th and July 8th) and three times in 2022 (June 17th, 28th and July 11th) using 1 to 9 scale, where 9 represents highest infection. The values were later converted into percentage scale, where 1 score is 0%, 1.5 = 2.5%, 2 = 5%, 2.5 = 7.5%, 3 = 10%, 3.5 = 15%, 4 = 20%, 4.5 = 30%, 5 = 40%, 5.5 = 50%, 6 = 60%, 6.5 = 65%, 7 = 70%, 7.5 = 75%, 8 = 80%, 8.5 = 85%, 9 = 90% for analysis.

Field trials of the NMBU spring wheat panel (Lin et al. 2022) used for validation were conducted in Norway in year 2018 in Sande (59°35′ N, 10°12′ E), Vestfold (Sa18) and Staur (St18), while in year 2019 at Vollebekk (Vb19), Staur (St19) and Holmestrand (59°29′ N, 10°14′ E), Vestfold (Hs19) (Fig.S1).

### Seedling inoculation

A subset of 25 genotypes from the NOBALwheat panel was chosen for seedling inoculations. These selected genotypes were subsequently inoculated with 15 different powdery mildew isolates of European origin. Among these genotypes, five lines carried the susceptibility haplotype “G_C” of *QPm.NOBAL-3A*, while the remaining 20 lines carried the resistance haplotype “A_C.”

The virulence to race-specific resistance genes of powdery mildew isolates was evaluated on a set of 13 differential lines (Table S1) obtained from the genebank of the Centre of Estonian Rural Research and Knowledge (https://nordic-baltic-genebanks.org/). The powdery mildew cultures were kept by regular passaging on wheat cultivar ‘Kanzler’, which was also used as a susceptible control in experiments. To determine the seedling resistance, selected wheat genotypes were subjected to powdery mildew challenge in an attached leaf assay. Plants were grown in a 7 × 7 cm pots filled with plant growth substrate under 16/8h (light/dark) cycles at 19 ± 2 °C under plastic cover, until the first leaf was expanded. Then the leaves were gently attached to acrylic glass panel by a double-sided sticky tape with adaxial side of the leaf facing outwards. The attached leaves were inoculated with powdery mildew 7–10 days after sowing. Even inoculation was achieved under a laminar flow hood by blowing conidiospores through a hole of a settling tower placed over the plants. Immediately after inoculation, the plants were covered by the cellophane cover to prevent cross-contamination. Inoculated plants were placed in a growth chamber under 16/8h (light/dark) cycles at 17°C for the development of phenotype. The resistance phenotype was scored visually 7–10 days after inoculation on 0–9 scale, where “0” means no signs of infection, “1” highly resistant and “9” means highly susceptible.

### Statistical analysis

The R packages “lsmeans” (Lenth 2016) and “lme4” (Bates et al. 2015) were utilized to correct for random block effects and calculate the least square mean of powdery mildew disease severity for each line in each disease trial. The pair-wise Pearson's correlation coefficients were calculated and visualized by R package “PerformanceAnalytics” (Peterson &Carl, 2020). Broad sense heritability was estimated by setting both genotypic and environmental effects as random effects, using the formula ${h}^{2} = {\sigma }_{g}^{2}/({\sigma }_{g}^{2}+{\sigma }_{E}^{2}/l+{\sigma }_{e}^{2}/l)$, where ${\sigma }_{g}^{2}$ is the genetic variance, ${\sigma }_{E}^{2}$ is the environment variance, and ${\sigma }_{e}^{2}$ is the error variance, l is the number of trials.

### Association and bioinformatic analysis

Association analyses were performed using the “BLINK” model with principal components as covariates (Huang et al. 2019) in R package “GAPIT3” (Wang & Zhang 2021). The Bonferroni threshold was calculated using the function “CalcThreshold” implemented in R package “RAINBOWR” (Hamazaki and Iwata 2020) to determine significant markers based on -log10(p) values. The software “TASSEL 5” (Bradbury et al. 2007) was used for calculating the pairwise linkage disequilibrium (LD) of markers on each chromosome with the full-matrix option. To summarize the relationship between LD decay and physical map distance, a non-linear model was employed, as described by Marroni et al. (2011). The half decay distance was subsequently calculated based on the estimated maximum value of LD. Marker sequences were obtained from databases https://triticeaetoolbox.org and http://www.cerealsdb.uk.net. The physical map positions of SNP markers on the ‘Chinese Spring’ wheat reference genome IWGSC RefSeq v1.0 (International Wheat Genome Sequencing et al., 2018) were obtained from the database https://urgi.versailles.inra.fr/blast/?dbgroup = wheat_iwgsc_refseq_v1_chromosomes&program = blastn. Significant markers located within the half decay distance were considered as belonging to the same QTL. The expressions of the genes within ± 0.5 Mbp interval of the most significant marker *AX-94555538* of *QPm.NOBAL-3A* were compared through “WheatOmics” database (Ma et al. 2021) with the data from a transcriptome study of powdery mildew infected wheat by Zhang et al. (2014).

### Haplotype analysis, KASP genotyping, and allele stacking

The haplotype analysis of *QPm.NOBAL-3A* was performed using the significant markers *AX-94555538* (8.3 Mbp) and *RFL_Contig1488_671* (8.7 Mbp). The powdery mildew disease severity (%) was compared pairwise using the Wilcoxon test and visualized with the R package “ggpubr” (Kassambara 2020). Both markers, *AX-94555538* and *RFL_Contig1488_671,* were converted to KASP markers. Marker primer sequences were obtained from CerealsDB (https://www.cerealsdb.uk.net/cerealgenomics/CerealsDB/) (Table S2).

For the allele stacking analysis, four significant markers from the consistent QTL across countries were selected, which explained more than 5% of the phenotypic variations in at least one environment (Table 1). Resistance alleles were obtained by comparing the best linear unbiased estimates (BLUEs) of disease severity for each line in all trials using the Wilcoxon test (Fig. S2), and lines with the same number of resistance alleles were grouped together. Differences in BLUEs between groups were tested using the Tukey’s HSD test (*P* < 0.05) with the R package “multcomView” (Graves et al. 2015).

**Table 1:** Significant markers used for allele stacking analysis

Marker	QTL	Chromosome	Physical position (Mbp)	Alleles (Resistance/Susceptibility)	Mean of Powdery mildew resistance for each allele (%)
*GENE-0918_159*	*QPm.NOBAL-2B*	2B	783.4	A/G	12.3/15.0
*AX-94555538*	*QPm.NOBAL-3A*	3A	8.3	A/G	13.0/23.9
*Excalibur_c99101_82*	*QPm.NOBAL-5A.1*	5A	403.9	A/G	13.6/24.0
*Excalibur_c26671_57*	*QPm.NOBAL-5A.2*	5A	591.3	C/T	12.3/20.9

## Results

### Phenotypic variation

**Fig. 1:**
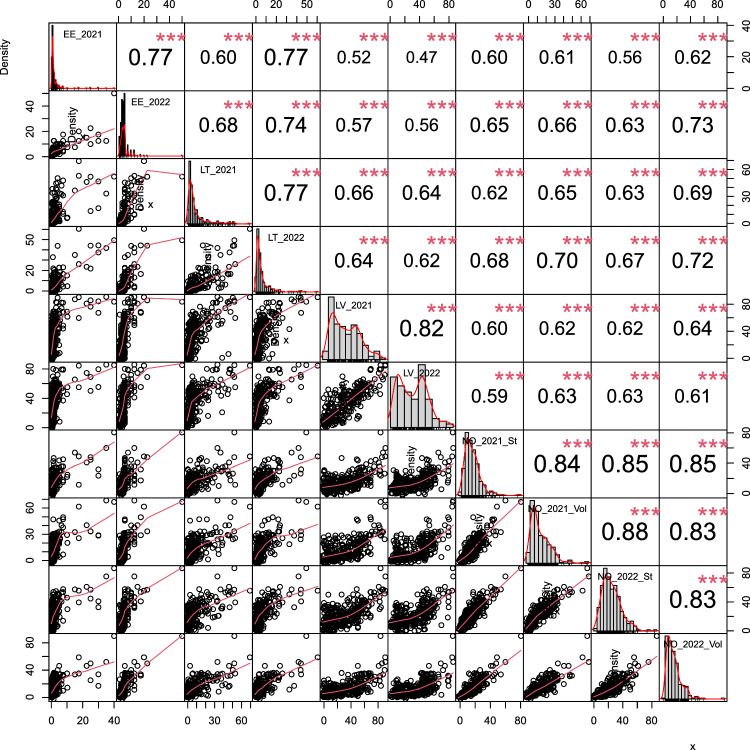
Comparative Analysis of Powdery Mildew Severity Across Trials. Histograms of powdery mildew severity in each trial are shown on the diagonal. The Pearson's correlations of the powdery mildew severity between trials are shown on the top of the diagonal, ***: *P* < 0.001. The bivariate scatter plots with a fitted line are shown on the bottom of the diagonal (EE: Estonia; LT: Lithuania; LV: Latvia; St: Staur, Norway; Vol: Vollebekk, Norway)

In order to get comparable data with the best differentiation between resistant and susceptible lines from each trial, the third scoring in 2021 and the fourth scoring in 2022 were selected as phenotypic input from the trials in Estonia. Significant variations in disease severities were observed at several time points in Latvia and Lithuania. Therefore, the average scores of two to three time points were selected as phenotypic input for these two locations, while for the Norwegian trials, the single scores at the point of maximum disease differentiation were used. In general, the resulting phenotypic distributions were right skewed; however, they were continuously distributed and close to normality (Fig. 1). The majority of lines exhibited powdery mildew disease severity below 40%. Despite the relatively lower disease pressure in Estonia and Lithuania as compared to the trials carried out in Norway and Latvia, significant correlations between all tested trials were observed, with Pearson's correlation coefficients ranging from 0.47 to 0.88 (Fig. 1). As expected, the correlation coefficients of trials within the same country were higher than those from different countries (Fig. 1). The broad-sense heritability of powdery mildew scores was estimated to be 0.83.

### Linkage disequilibrium and association analysis

The estimated r^2^ of half decay (critical threshold of r^2^) for the panel was 0.23, and the estimated physical half decay distance was 2.69 Mbp. After adjusting for multiple testing, the Bonferroni threshold for our dataset was determined to be -log10(p) = 5.67. Through principal component analysis (PCA), the NOBAL wheat panel can be separated into 5 subpopulations (Fig. S3). The first four PCs were used as covariates by the BLINK model in the GAPIT3 R package (Wang and Zhang 2021), and 85 significant marker-trait associations (MTAs) were identified. Among these, we detected 11 consistent QTL on wheat chromosomes 1A, 1B, 2A, 2B, 3A, 3B, 5A, 6A, 6B, and 7B across environments (Table 2). Of these, six QTL were identified across environments within a country, while five were identified across environments across countries (Table 2).

**Table 2:** Consistently significant QTL of powdery mildew resistance across environments (EE: Estonia; LT: Lithuania; LV: Latvia; St: Staur, Norway; Vol: Vollebekk, Norway)

QTL	Chromosome	Position (Mbp)	Trial	− log10(p)	Phenotypic variance explained (%)	Reference
*QPm.NOBAL-1A*	1A	13.8	NO_2021_St, NO_2022_Vol	6.18–6.22	1.71–3.32	Koller et al. 2018
*QPm.NOBAL-1B*	1B	665.3	NO_2021_St, NO_2022_St	6.02–10.38	3.50–5.89	Lillemo et al. 2008
*QPm.NOBAL-2A*	2A	3.8–4.2	EE_2022, LV_2022	5.72–5.78	0.06–3.18	This study
*QPm.NOBAL-2B*	2B	780.8–783.4	LT_2021, NO_2022_St	7.99–9.93	2.30–9.99	Yin et al. 2009; Tan et al. 2019
*QPm.NOBAL-3A*	3A	8.3–8.7	LT_2021, NO_2021_Vol, EE_2021, EE_2022,	6.49–9.59	1.41–38.30	Lillemo et al. 2008; Yang et al. 2017
*QPm.NOBAL-3B*	3B	55.46	NO_2021_St, NO_2022_Vol	7.68–12.81	1.79–4.14	This study
*QPm.NOBAL-5A.1*	5A	403.9	LV_2022, NO_2021_St	7.42–9.22	9.50–14.43	This study
*QPm.NOBAL-5A.2*	5A	591.1–591.3	LV_2021, NO_2021_St	9.02–13.92	3.26–9.67	Alemu et al. 2021
*QPm.NOBAL-6A*	6A	604.6–605.2	NO_2021_St, NO_2022_St	6.24–11.57	2.18–15.40	This study
*QPm.NOBAL-6B*	6B	699.01	LV_2021, LV_2022	5.69–11.39	4.42–32.27	Hinterberger et al. 2022
*QPm.NOBAL-7B*	7B	572.4	EE_2021, EE_2022	6.99–7.91	2.59–3.11	Alemu et al. 2021

One notable QTL, *QPm.NOBAL-2A*, was consistently detected in Estonia and Latvia, spanning the region of 3.8–4.2 Mbp on the short arm of chromosome 2A. This QTL explained up to 3.2% of the phenotypic variation. Another QTL, *QPm.NOBAL-2B*, exhibited consistent associations across Lithuania and Norway, mapping to the long arm of chromosome 2B (780.8–783.4 Mbp) and explaining from 2.3% to 10% of the phenotypic variation. Notably, *QPm.NOBAL-3A* emerged as the most robust QTL, being significantly detected in four trials conducted in three different countries. This QTL was located on the short arm of chromosome 3A (8.3–8.7 Mbp), with − log10(p) values ranging from 6.49 to 9.59, and explained up to 38.3% of the phenotypic variation. Two additional QTL, *QPm.NOBAL-5A.1* and *QPm.NOBAL-5A.2*, were consistently identified on the long arm of chromosome 5A, at positions 404 Mbp and 591 Mbp, respectively. *QPm.NOBAL-5A.1* was identified in the Staur trial conducted in Norway in 2021 and the trial in Latvia in 2022, while *QPm.NOBAL-5A.2* was detected in trials conducted in Staur, Norway, and Latvia in 2021.

Furthermore, we identified four QTL that were specific to Norwegian environments. *QPm.NOBAL-1A* was detected in the Staur trial in 2021 and the Vollebekk trial in 2022, located at 13.8 Mbp on chromosome 1A. *QPm.NOBAL-1B* was identified in the Staur trials of both 2021 and 2022, mapping to 665.3 Mbp on chromosome 1B and explaining phenotypic variations ranging from 3.5% to 5.9%. *QPm.NOBAL-3B* was detected in the Staur trial of 2021 and the Vollebekk trial of 2022, positioned at 55.46 Mbp on chromosome 3B. Additionally, *QPm.NOBAL-6A* was identified on chromosome 6A, spanning the region of 604.6–605.2 Mbp, and accounted for phenotypic variations of up to 15.4%. Moreover, we discovered one QTL specific to Latvia and one specific to Estonia across years. *QPm.NOBAL-6B* was detected in Latvian trials conducted in both 2021 and 2022, mapping to the long arm of chromosome 6B (699.01 Mbp) with -log10(p) values ranging from 5.69 to 11.39. *QPm.NOBAL-7B* was identified exclusively in Estonian trials, located at 572.4 Mbp on the long arm of chromosome 7B, and explained phenotypic variations ranging from 2.59% to 3.11%.

### Haplotype analysis

**Fig. 2:**
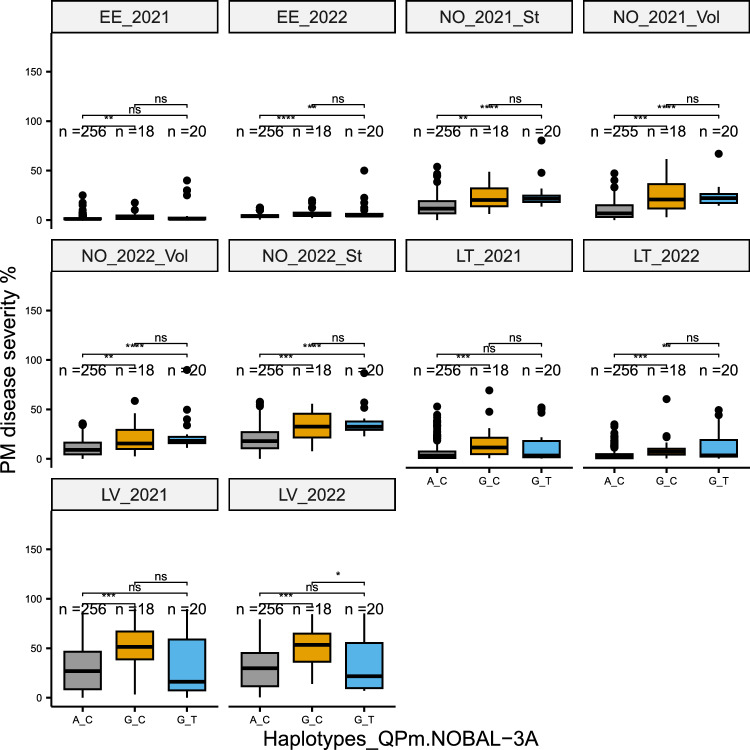
Haplotype analysis of *QPm.NOBAL-3A* in the NOBALwheat panel (EE: Estonia; LT: Lithuania; LV: Latvia; St: Staur, Norway; Vol: Vollebekk, Norway). Differences in powdery mildew severity (%) between haplotypes were determined by the Wilcoxon test. ns: *P* > 0.05; *: *P* < 0.05; **: *P* < 0.01; ***: *P* < 0.001; ****: *P* < 0.0001

According to Table 2, *QPm.NOBAL-3A* was the most consistent QTL across environments; thus, it was selected for further haplotype analysis. As shown in Fig. 2, genotypes in the NOBALwheat panel were grouped into three haplotypes based on different combinations of alleles of the two markers at this QTL, namely *AX-94555538* and *RFL_Contig1488_671*. The majority of genotypes (87.1%) belong to the “A_C” haplotype, whereas only 6.1% of lines have haplotype “G_C” and 6.8% of lines have haplotype “G_T.” Interestingly, consistent differences in disease severity were observed between haplotype “A_C” and “G_C” in all tested trials, with “A_C” always showing lower severity in comparison with haplotype “G_C” (Fig. 2). The haplotype effect varied from 2.3% to 21.9%, depending on the disease pressure of the individual trial. In six out of the ten tested trials, significant differences in disease severity were also detected between haplotype “A_C” and “G_T”, with “G_T” showing higher severity compared to “A_C.” A significant difference between haplotype “G_C” and “G_T” was only observed in trial 2022, Latvia. Thus, our results illustrated that “A_C” was the resistance haplotype, while “G_C” and “G_T” were susceptibility haplotypes. In other words, alleles of the first marker *AX-94555538* were more crucial for explaining the phenotypic effects of this locus*.*

**Fig. 3:**
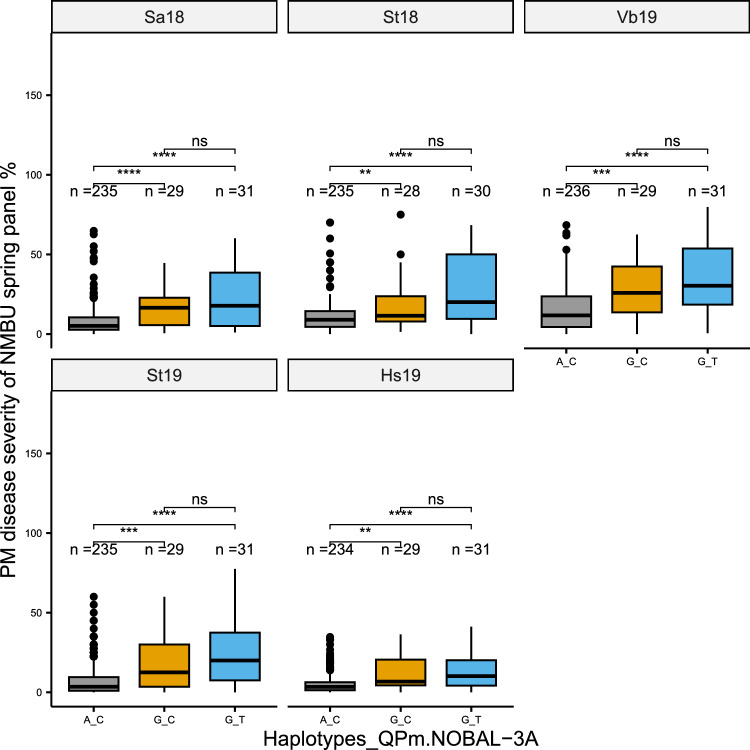
Haplotype analysis of *QPm.NOBAL-3A* in the NMBU spring wheat panel (Lin et al. 2022), (Sa: Sande, Norway; St: Staur, Norway; Vb: Vollebekk, Norway; Hs: Holmestrand, Norway). Differences in powdery mildew severity (%) between haplotypes were determined by the Wilcoxon test. ns: *P* > 0.05; **: *P* < 0.01; ***: *P* < 0.001; ****: *P* < 0.0001

To verify the haplotype effects of QTL *QPm.NOBAL-3A*, the same haplotype analysis was conducted using powdery mildew field data of the NMBU spring wheat panel, which was tested in five environments in Norway from 2018 to 2019 (Fig. 3). Significant differences between haplotypes “A_C” and “G_C”, “A_C” and “G_T” were detected in all tested environments, while no significant difference was found between the susceptibility haplotypes “G_C” and “G_T” (Fig. 3). In accordance with the haplotype results using NOBALwheat panel, “A_C” was the resistance haplotype, and “G_C” and “G_T” were susceptibility haplotypes (Fig. 3). Validation of KASP markers was performed using the NMBU spring wheat panel, demonstrating consistent concordance between KASP genotyping results and SNP chip data (Table S2, Fig. S4).

### Seedling inoculation

**Fig. 4:**
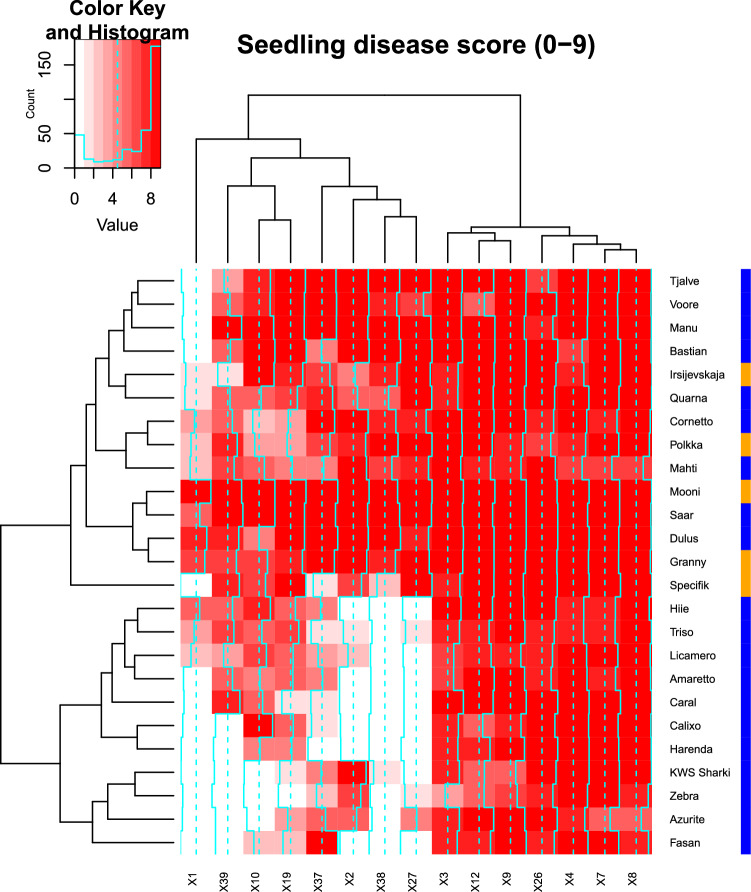
Heatmap showing the seedling reactions of 25 genotypes in NOBALwheat panel inoculated by 15 powdery mildew isolates. Each row corresponds to a genotype, and each column represents an isolate. The color intensity in each cell reflects the seedling reactions with a 0–9 scale, where 0 represents complete resistance without any symptoms, while 9 represents highly susceptible reaction. The genotype and isolates were hierarchically clustered based on the disease reaction patterns. The scale bar on the top left indicates the color range corresponding to the disease reaction. The vertical color bar on the right hand indicates the *QPm.NOBAL-3A* haplotype of the genotype, where orange is the susceptibility haplotype “G_C”, and blue indicates the resistance haplotype “A_C”

The heatmap in Fig. 4 illustrates the seedling disease scores of the 25 cultivars in the NOBALwheat panel inoculated with 15 different powdery mildew isolates. Notably, all genotypes displayed highly susceptible responses to isolates X3, X4, X7, X8, X9, X12, and X26, while exhibiting varying levels of disease responses to isolates X1, X2, X10, X19, X27, X37, X38 and X39. Interestingly, there was no clear indication that genotypes carrying the resistance haplotype of *QPm.NOBAL-3A* possessed a shared race-specific resistance gene. For instance, the CIMMYT line ‘Saar’, which showed considerable field resistance and carried the resistance haplotype of *QPm.NOBAL-3A*, proved susceptible to most of the isolates tested in this study at the seedling stage. Conversely, the cultivar ‘Spécifik’ carrying the susceptibility haplotype of *QPm.NOBAL-3A*, exhibited seedling resistance to isolates X1, X37 and X38.

### Stacking of resistance alleles

**Fig. 5:**
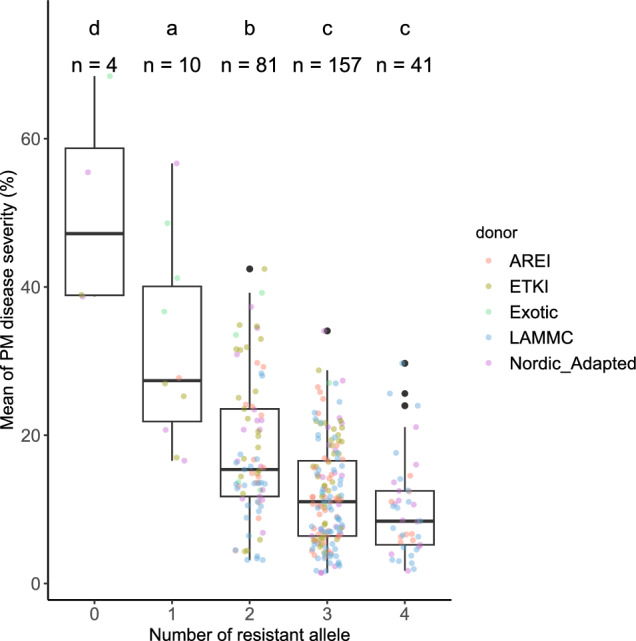
Boxplots showing the mean disease severities of lines grouped by the number of resistance alleles they carry from the four commonly identified QTL across multiple countries. Significant differences (*p* < 0.05) between groups are indicated by different letters above the boxplots, as determined by Tukey’s HSD test. Genotypes were color coded by the donor partner

The NOBALwheat panel exhibited genetic diversity, resulting in the classification of the panel into five groups based on the number of resistance alleles present for four consistent QTL across environments and countries in this study (Fig. 5). An evident correlation was observed, wherein a higher accumulation of resistance alleles corresponded to a notable reduction in disease severity (Fig. 5). Significantly different disease severities were observed among groups with varying numbers of resistance alleles, except for groups four and five, which displayed relatively low disease severities. Therefore, no statistical difference was detected between group four (carrying three resistance alleles) and group five (carrying four resistance alleles). Figure 5 also highlights that the exotic lines included in this panel consistently resulted in higher disease severities compared to the adapted lines or new breeding materials. Notably, there were four lines lacking any resistance alleles. The line that exhibited the highest disease severity among all lines was the Australian line ‘Avocet YrA’, which serves as a susceptible control for powdery mildew resistance testing. The old Norwegian cultivar ‘Reno’ and the breeding line ‘MS 273–150’, both from the 1970s, were also classed to this group and ‘MS 273–150’ is commonly used as moderately susceptible control for powdery mildew in Norway. ‘Reno’ is known to carry the *Pm4b* resistance gene, which is no longer effective in Norway (Hysing et al. 2007; Lillemo & Dieseth 2011). Additionally, the Finnish-Estonian cultivar ‘Mooni’ was also assigned to the same group, which was commonly used as susceptible control in Estonia. Ten lines were categorized into groups containing only one of the resistance alleles, with three of them originating from CIMMYT or China, representing exotic germplasm. These exotic lines exhibited a lower number of resistance alleles, resulting in higher disease severities when challenged by powdery mildew, in contrast to the Nordic adapted lines and Baltic breeding lines (Fig. 5). To further investigate the relationship between the year of release for cultivars and year of hybridization for breeding lines, source of the lines, and the number of resistance alleles, we conducted an analysis excluding the exotic lines from the panel (Fig. S5). Although the majority of genotypes in the NOBALwheat panel were relatively new lines released after 2010, in most cases, a clear trend could still be observed that the mean powdery mildew disease severity decreased in more recent lines compared to older germplasms (Fig. S5).

## Discussion

A significant proportion of the NOBALwheat panel comprises relatively recent cultivars and breeding lines. Consequently, a substantial number of lines exhibited high resistance against powdery mildew within the panel (Fig. 1). This indicates efficiency of wheat breeding for powdery mildew resistance in Nordic and Baltic breeding programs and gradual loss of resistance in old genotypes which relied mostly on single race-specific *Pm* genes which were combated by the pathogen evolution. While most lines originated from domestic sources, a small percentage (3%) were sourced from exotic origins. The exotic lines displayed highly susceptible responses to the disease in general, particularly the lines from CIMMYT. Nonetheless, we successfully identified several key QTL, which can be harnessed for marker-assisted selection to select against the undesirable alleles. The QTL identified across various environments and countries can be potentially durable QTL with a broad spectrum of resistance.

Upon comparing the significant QTL identified in our study with previously reported QTL, a notable overlap was observed, as several QTL were found to be mapped to positions that coincide with the previously reported loci of powdery mildew resistance genes/QTL. For instance, *QPm.NOBAL-1A* was identified on the short arm of chromosome 1A (13.8 Mbp) and was only detected in the Norwegian environments. It might collocate with the race-specific resistance gene *Pm3*, which was mapped to 4.5 Mbp on chromosome 1A (Koller et al. 2018). Another Norwegian environment-specific QTL *QPm.NOBAL-1B* (665.3 Mbp) mapped close to the pleiotropic resistance gene *Pm39/ Lr46/Yr29* (Lillemo et al. 2008) on the long arm of chromosome 1B at 662–665 Mbp. The resistance source of *Pm39/ Lr46/Yr29* was carried by the CIMMYT line ‘Saar’ (Lillemo et al. 2008), which was also included in the NOBALwheat panel. However, the genotyping for the significant marker *AX-158544942* of *QPm.NOBAL-1B* failed for ‘Saar’ in the NOBALwheat panel, and thus further validation was not possible. Moreover, *QPm.NOBAL-2B* mapped to the distal end of chromosome 2B at position 780.8–783.4 Mbp. Tan et al. (2019) characterized and mapped the resistance gene *Pm63* on the distal end of chromosome 2B, which showed a wide spectrum of powdery mildew resistance. However, *Pm63* was mapped to 710–723 Mbp, which was around 60 Mbp away from *QPm.NOBAL-2B*. Thus, *QPm.NOBAL-2B* and *Pm63* were likely different QTL. However, Tan et al. (2019) also discussed another QTL *PmJM22* located on the long arm of 2B, identified by Yin et al. (2009). *PmJM22* was carried by a Chinese winter wheat cultivar ‘Jimai 22’ and linked to the SSR marker *Xwmc149* (779.1 Mbp) (Yin et al. 2009; Tan et al. 2019), which might be the same QTL as *QPm.NOBAL-2B* (780.8–783.4 Mbp). *QPm.NOBAL-5A.2,* mapped to 591 Mbp of the chromosome 5A might collocate with the adult plant resistant QTL by a genome-wide association study by Alemu et al. (2021), in which they mapped a QTL on chromosome 5A spanning the region of 607.7–607.8 Mbp. Additionally, *QPm.NOBAL-6B* was identified only by trials in Latvia (699 Mbp) and mapped close to a seedling resistance QTL at 697.6 Mbp by a recent study (Hinterberger et al. 2022). To our knowledge, *QPm.NOBAL-2A, QPm.NOBAL-3B, QPm.NOBAL-5A.1* and *QPm.NOBAL-6A* are novel QTL with no previous reports on wheat powdery mildew resistance on those chromosome regions.

*QPm.NOBAL-3A* was the most consistent QTL detected in our study, exhibiting a significant contribution to powdery mildew resistance by explaining up to 38.3% of the phenotypic variation. The robustness of the QTL was further demonstrated by the consistency of the significant haplotype effects observed in the validation panel, especially since the field data in the validation panel was obtained from different years and genotypes than the field trials of the NOBALwheat panel. The concordance between the results obtained from the NOBALwheat trials and the validation panel strengthens the confidence in the QTL’s performance and reinforces its potential for practical application in breeding programs for improving powdery mildew resistance in wheat cultivars. When comparing with other published powdery mildew QTL, it appears that *QPm.NOBAL-3A* aligns closely with the race non-specific resistance QTL on 3AS reported by Lillemo et al. (2008). They mapped the 3AS QTL to 7.2 Mbp on the short arm of chromosome 3A, which was close to the interval 8.3–8.7 Mbp region where *QPm.NOBAL-3A* was localized. The resistance source of the 3AS QTL derived from the cultivar ‘Saar’ (Lillemo et al. 2008), was confirmed to carry the resistance haplotype “A_C” of *QPm.NOBAL-3A*. Furthermore, the greenhouse inoculation experiments provided additional evidence that no common race-specific resistance pattern was detected among lines carrying the resistance haplotype. Rather, these lines displayed a high degree of susceptibility at the seedling stage while showing resistance in the field, which is a common feature of race non-specific adult plant resistance genes. These findings indicate that the casual resistance gene of *QPm.NOBAL-3A* is likely of race non-specific nature.

We explored transcriptome data of genes located within the ± 0.5 Mbp interval of the most significant marker of the *QPm.NOBAL-3A* QTL from Zhang et al. (2014) which involved sampling powdery mildew-infected wheat seedlings at various time points. By comparing this dataset using the “WheatOmics” database (Ma et al. 2021), we identified that *TraesCS3A02G009000* could serve as a candidate gene for *QPm.NOBAL-3A*, since this gene exhibited a rapid increase in expression levels at 24 h post-inoculation, followed by a decline at 48 h post-inoculation, yet maintaining higher expression compared to the non-inoculated control (Fig S6). According to the gene annotation (iwgsc_refseq_v1.1) (International Wheat Genome Sequencing et al., 2018), *TraesCS3A02G009000* was predicted to encode a serine-threonine/tyrosine-protein kinase, which plays a pivotal role in the recognition of pathogens and the activation of disease resistance mechanisms (Shi et al. 2016; Bhatia et al. 2021). This altered gene expression pattern likely resulted from pathogen infection; however, further validation through comparative transcriptome analyses on lines carrying resistance and susceptibility haplotypes of *QPm.NOBAL-3A* will be required.

Pyramiding powdery mildew resistance genes/QTL in bread wheat is a promising approach to enhance resistance against this devastating fungal disease, as evidenced by the allele stacking analysis in our study. However, quite a few exotic powdery mildew resistance genes were derived from related cereal species or wild relatives, such as *Pm20* from *Secale cereale* (Friebe et al. 1994), *Pm25* from *Triticum monococcum* (Shi et al. 1998), *Pm35* from *Aegilops tauschii* (Miranda et al. 2007), *Pm37* from *Triticum timopheevii* (Perugini et al. 2008), and *Pm60* from *Triticum urartu* (Zou et al. 2018), of which utilization in bread wheat usually require intensive efforts to mitigate the linkage drag of undesired traits. An elite wheat panel was employed in our study, revealing that a substantial proportion of resistance alleles had already been introgressed into the germplasm. Rather than introducing additional exotic materials to enhance powdery mildew resistance, a more efficient approach may involve marker-assisted selection to identify and eliminate susceptibility alleles. The identification and validation of robust QTL in this study, along with the availability of closely linked markers, offer valuable tools for breeders to precisely select and pyramid resistance alleles. Such approaches are needed to facilitate the development of powdery mildew resistant wheat varieties for the changing climate.

### Supplementary Information

Below is the link to the electronic supplementary material.

Fig. S1 Geographic distribution of powdery mildew field trials for the NOBALwheat mapping panel and NMBU validation panel. (NW: NOBALwheat mapping panel; EE: Estonia; LT: Lithuania; LV: Latvia; St: Staur, Norway; Vol: Vollebekk, Norway, Sa: Sande, Norway; Hs: Holmestrand, Norway) (TIF 4620 KB)

Fig. S2 Allele effect of markers used for allele stacking analysis. Differences in powdery mildew severity (%) between alleles of the marker were determined by the Wilcoxon test. *: P < 0.05; ****: P < 0.0001. (PDF 7 KB)

Fig. S3 Principal component analysis (PCA) eigenvalue plot of the NOBALwheat panel using 18562 SNP markers. (PDF 5 KB)

Fig. S4 Genotype calls for allele one (FAM) is indicated in blue while allele two (HEX) in red. Heterozygous scores are shown in green, negative controls are in black, and uncertain scores in pink (treated as missing in genotyping results). (PDF 137 KB)

Fig. S5 Mean of PM disease severity of lines with different year of release grouped by donor partner. (PDF 21 KB)

Fig. S6 Expression of candidate genes of the QTL QPm.NOBAL-3A by dataset from Zhang et al., (2014). (PNG 69 KB)

Supplementary file7 (XLSX 47 KB)

Supplementary file8 (docx 15 KB)

## Data Availability

All the phenotype and genotype data used in this study are provided as supplementary files.
