# Supplementary figures and images for: Genome-wide association analysis identifies a consistent QTL for powdery mildew resistance on chromosome 3A in Nordic and Baltic spring wheat
Source: Theor Appl Genet. 2024 Jan 19;137(1):25. doi: 10.1007/s00122-023-04529-1 (PMC10799116; doi:10.1007/s00122-023-04529-1)

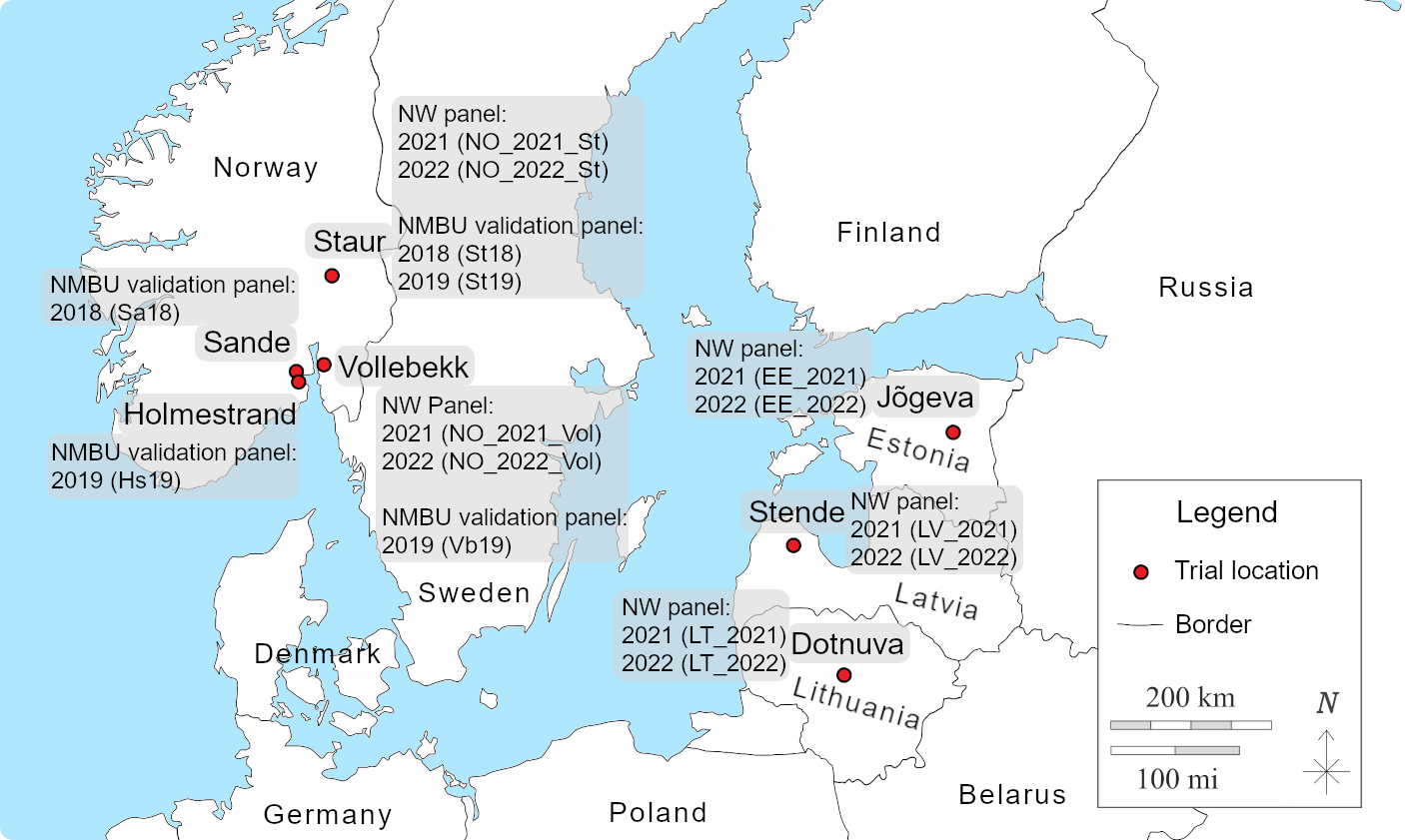

Supplement: Supplementary file 1 — Fig. S1 Geographic distribution of powdery mildew field trials for the NOBALwheat mapping panel and NMBU validation panel. (NW: NOBALwheat mapping panel; EE: Estonia; LT: Lithuania; LV: Latvia; St: Staur, Norway; Vol: Vollebekk, Norway, Sa: Sande, Norway; Hs: Holmestrand, Norway) (TIF 4620 KB) [file 122_2023_4529_MOESM1_ESM.tif]

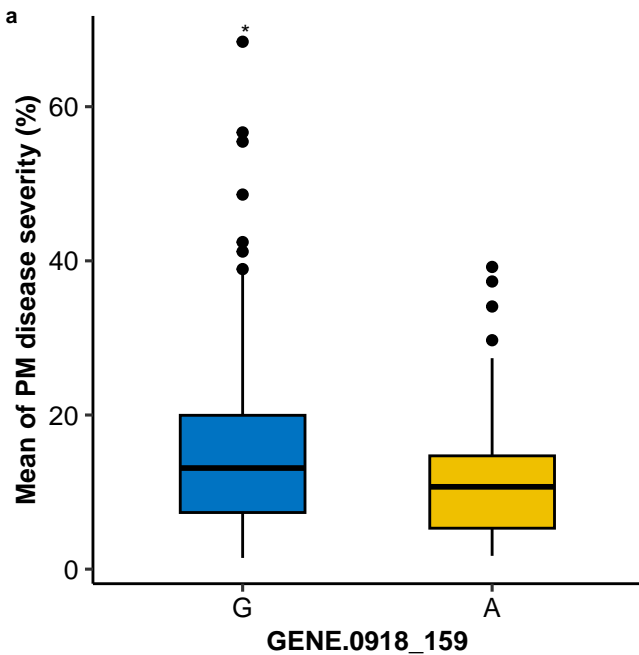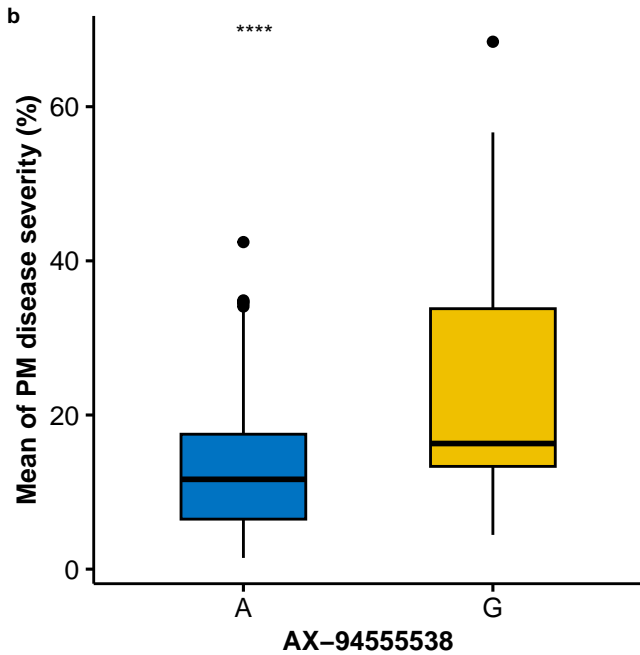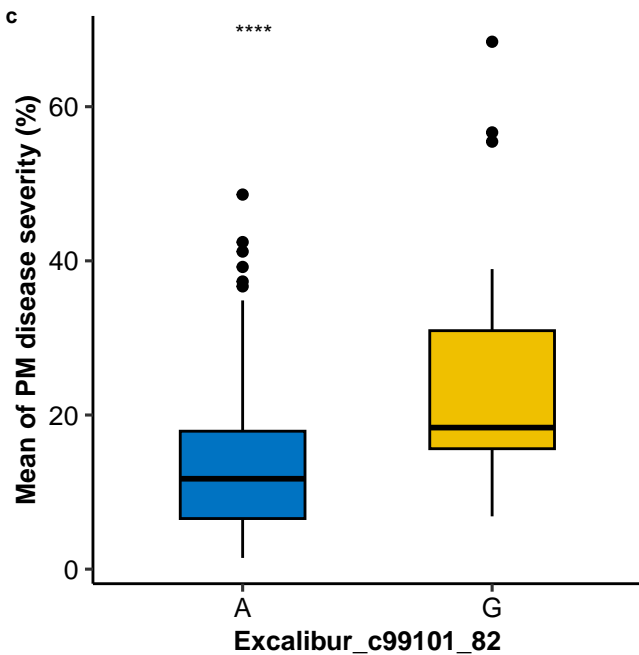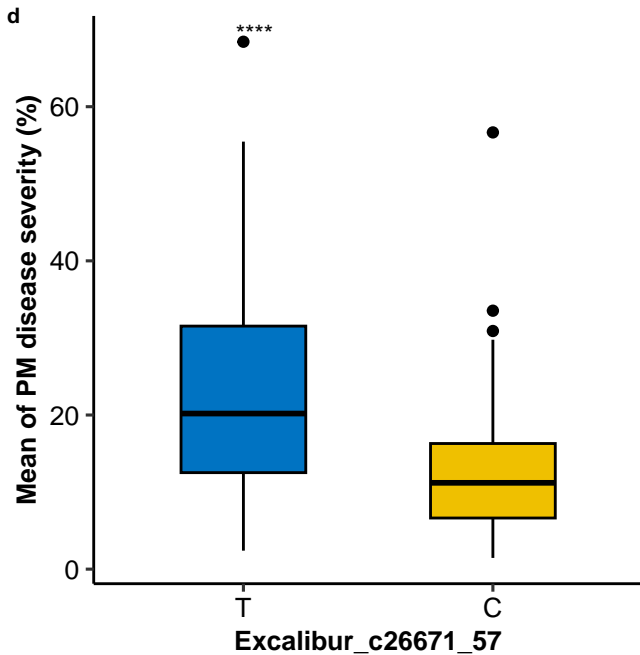

Supplement: Supplementary file 2 — Fig. S2 Allele effect of markers used for allele stacking analysis. Differences in powdery mildew severity (%) between alleles of the marker were determined by the Wilcoxon test. *: P < 0.05; ****: P < 0.0001. (PDF 7 KB) [file 122_2023_4529_MOESM2_ESM.pdf]

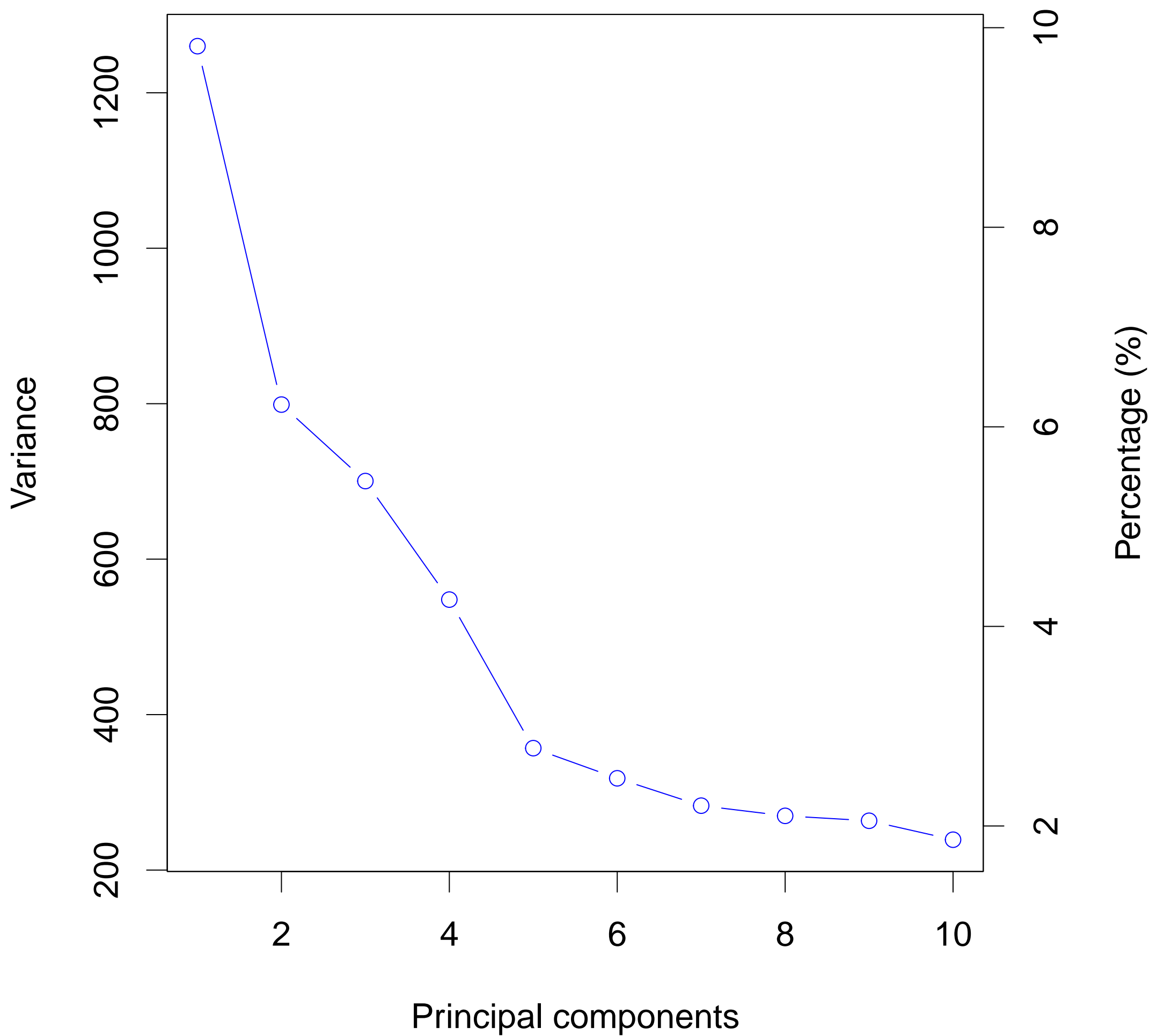

Supplement: Supplementary file 3 — Fig. S3 Principal component analysis (PCA) eigenvalue plot of the NOBALwheat panel using 18562 SNP markers. (PDF 5 KB) [file 122_2023_4529_MOESM3_ESM.pdf]

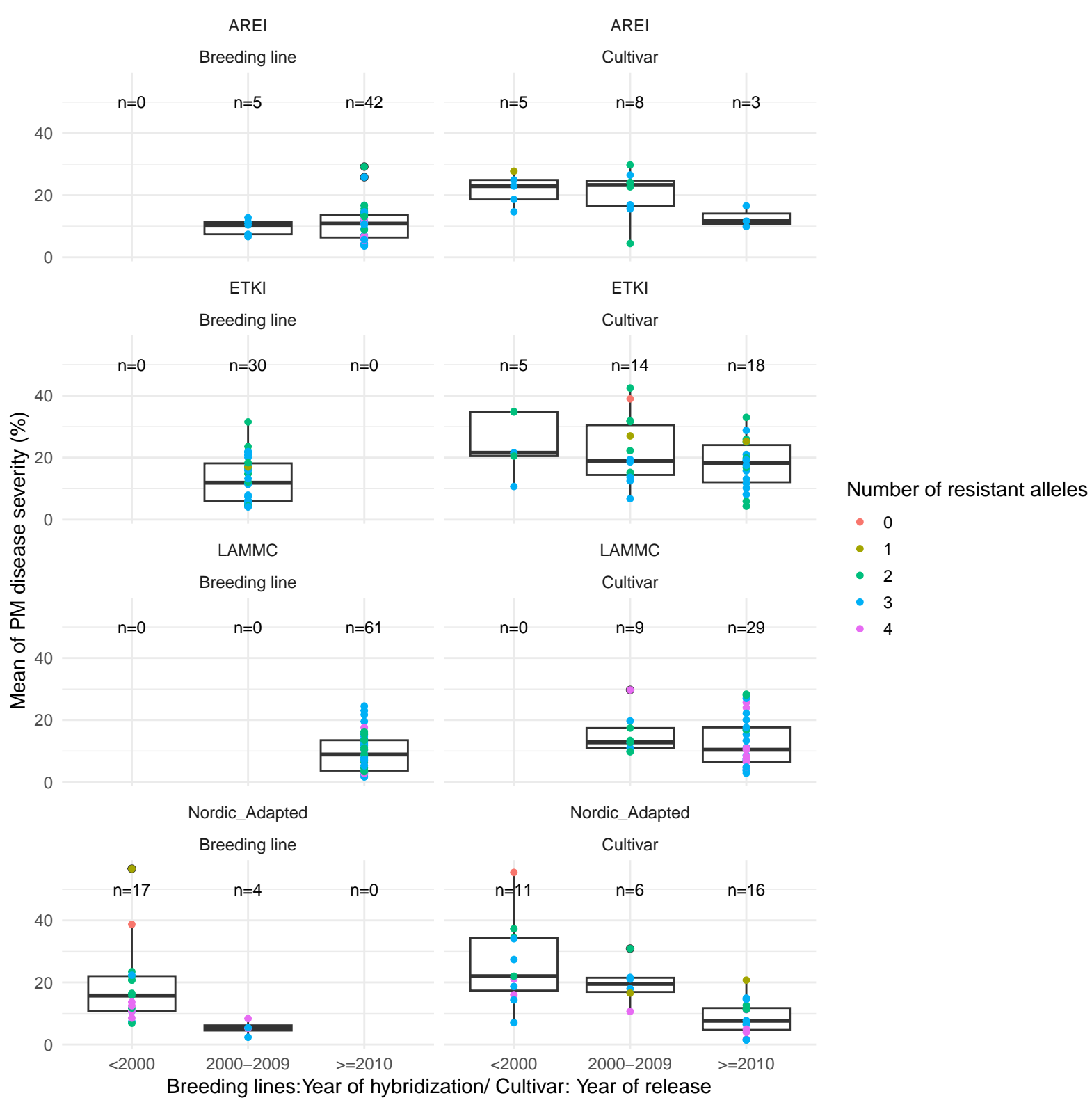

Supplement: Supplementary file 5 — Fig. S5 Mean of PM disease severity of lines with different year of release grouped by donor partner. (PDF 21 KB) [file 122_2023_4529_MOESM5_ESM.pdf]

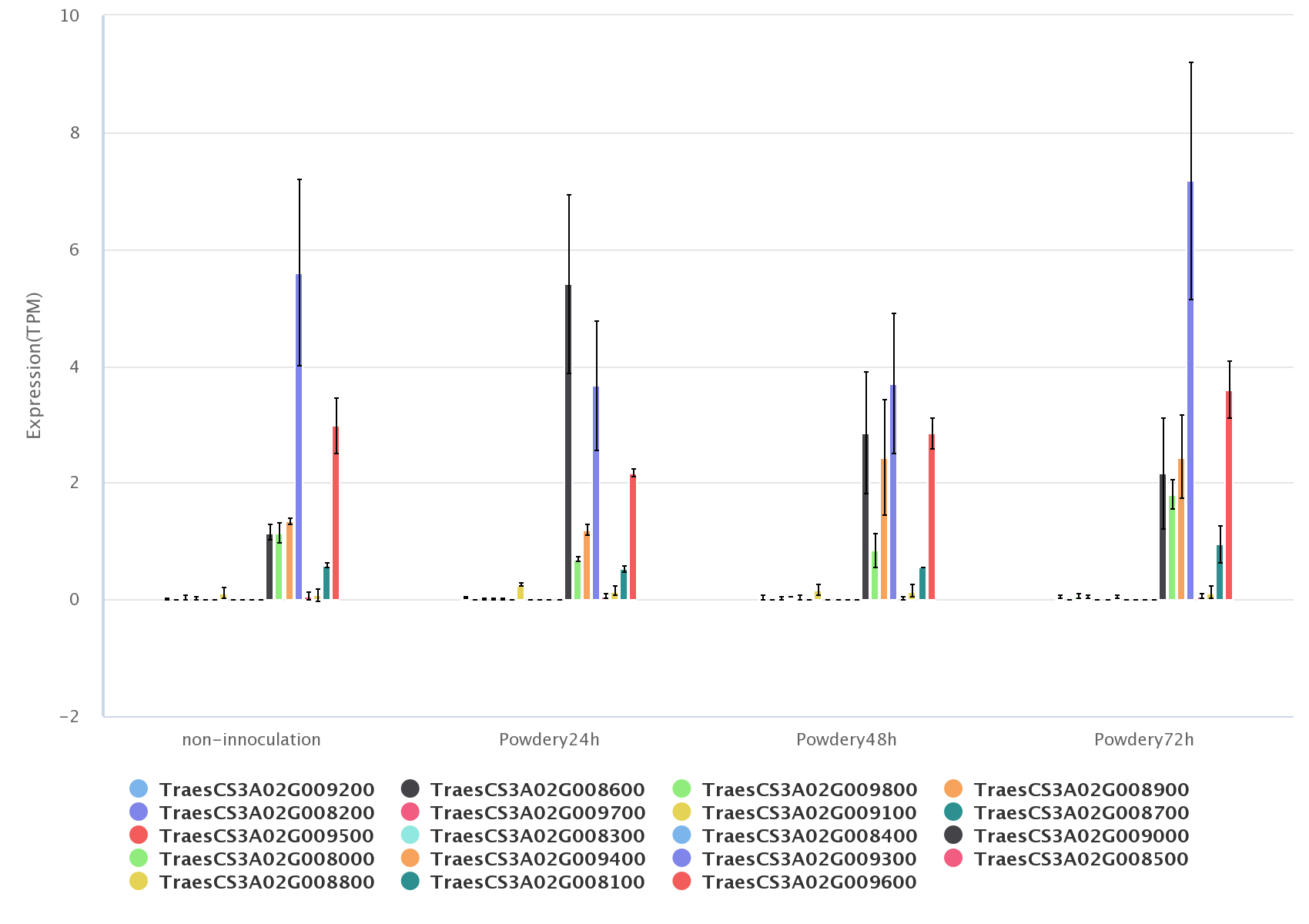

Supplement: Supplementary file 6 — Fig. S6 Expression of candidate genes of the QTL QPm.NOBAL-3A by dataset from Zhang et al., (2014). (PNG 69 KB) [file 122_2023_4529_MOESM6_ESM.png]
