# Supplementary material for: Genome-wide association analysis identifies a consistent QTL for powdery mildew resistance on chromosome 3A in Nordic and Baltic spring wheat
Source: Theor Appl Genet. 2024 Jan 19;137(1):25. doi: 10.1007/s00122-023-04529-1 (PMC10799116; doi:10.1007/s00122-023-04529-1)

AX-94555538

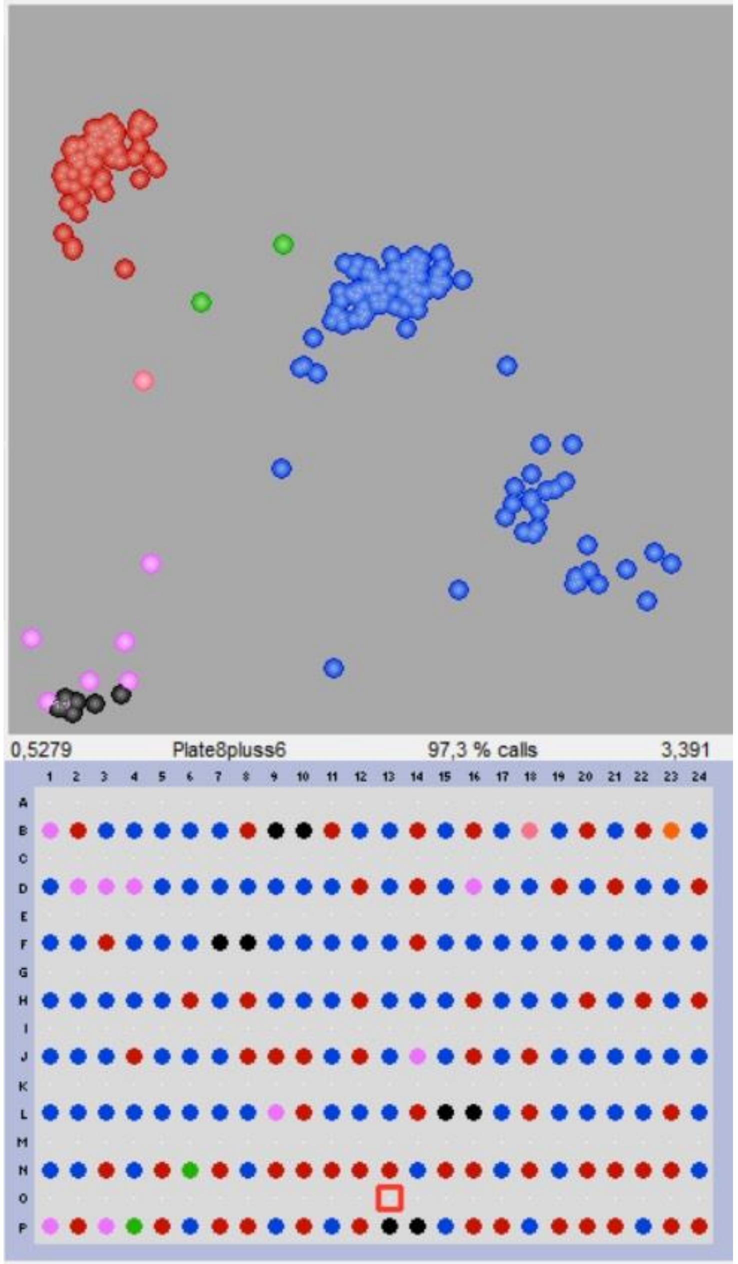

RFL_Contig1488_671

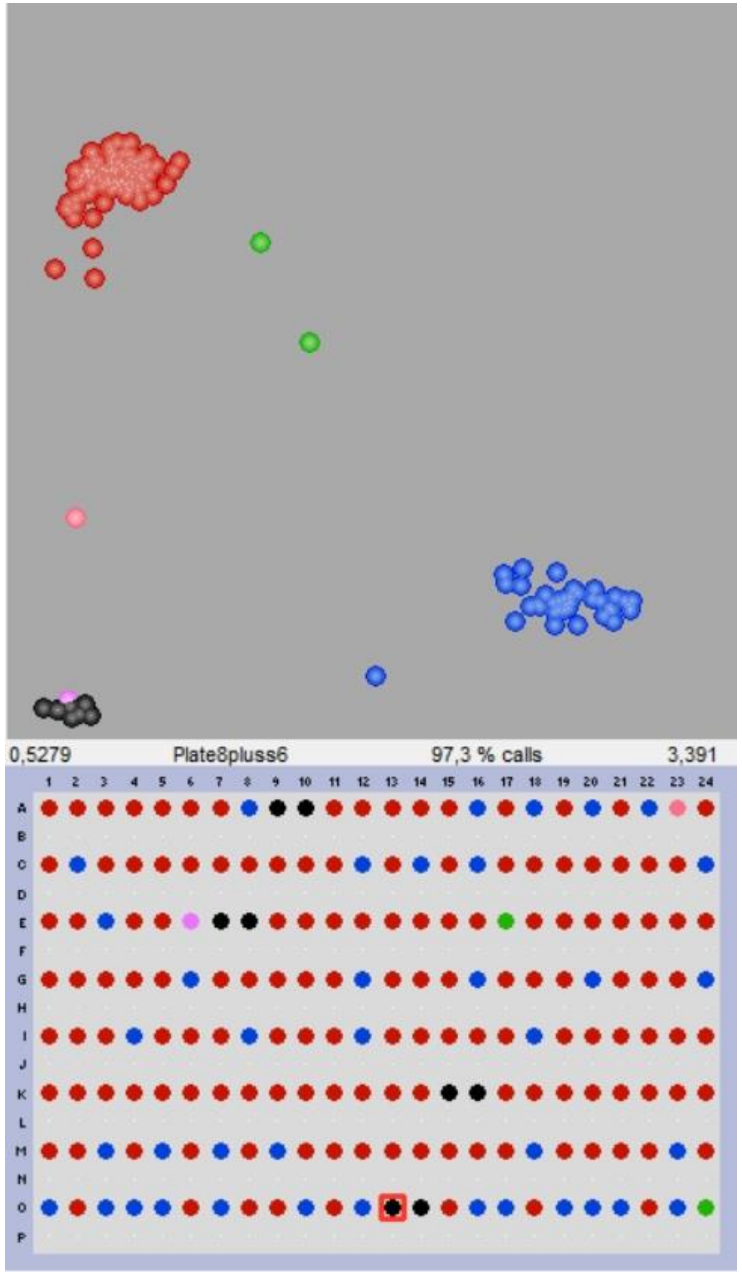

Supplement: Supplementary file 4 — Fig. S4 Genotype calls for allele one (FAM) is indicated in blue while allele two (HEX) in red. Heterozygous scores are shown in green, negative controls are in black, and uncertain scores in pink (treated as missing in genotyping results). (PDF 137 KB) [file 122_2023_4529_MOESM4_ESM.pdf]
