# Supplementary material for: Genome-wide association analysis identifies a consistent QTL for powdery mildew resistance on chromosome 3A in Nordic and Baltic spring wheat
Source: Theor Appl Genet. 2024 Jan 19;137(1):25. doi: 10.1007/s00122-023-04529-1 (PMC10799116; doi:10.1007/s00122-023-04529-1)
Supplement: Supplementary file 8 — Supplementary file8 (docx 15 KB) [file 122_2023_4529_MOESM8_ESM.docx]

Table S1 The virulence of 15 powdery mildew isolates on 13 differential lines with different race-specific resistance genes.

| Differential | Resistance gene | X1 | X39 | X10 | X19 | X37 | X2 | X38 | X27 | X3 | X12 | X9 | X26 | X4 | X7 | X8 |
| --- | --- | --- | --- | --- | --- | --- | --- | --- | --- | --- | --- | --- | --- | --- | --- | --- |
| Axminster/8*Chancellor | *Pm1a* | 4 | 9 | 9 | 7 | 5 | 1 | 2 | 4 | 8 | 7 | 8 | 9 | 8 | 9 | 8 |
| Ulka/8*Chancellor | *Pm2a* | 7 | 9 | 7 | 8 | 9 | 9 | 9 | 9 | 9 | 9 | 9 | 9 | 9 | 9 | 9 |
| Asosan/8*Chancellor | *Pm3a* | 2 | 4 | 7 | 6 | 6 | 9 | 4 | 9 | 1 | 9 | 9 | 3 | 1 | 3 | 3 |
| Chul/8*Chancellor | *Pm3b* | 1 | 3 | 9 | 4 | 4 | 9 | 6 | 9 | 1 | 6 | 3 | 3 | 2 | 2 | 2 |
| Sonora/8*Chancellor | *Pm3c* | 4 | 8 | 9 | 8 | 6 | 8 | 6 | 9 | 3 | 7 | 8 | 3 | 3 | 2 | 4 |
| Kolibri | *Pm3d* | 6 | 8 | 9 | 9 | 9 | 8 | 9 | 9 | 9 | 9 | 8 | 9 | 8 | 9 | 9 |
| Khapli/8*Chancellor | *Pm4a* | 1 | 8 | 9 | 8 | 8 | 9 | 7 | 9 | 9 | 9 | 9 | 9 | 9 | 9 | 9 |
| Armada | *Pm4b* | 2 | 8 | 9 | 9 | 9 | 9 | 8 | 8 | 9 | 9 | 9 | 9 | 7 | 8 | 9 |
| Hope | *Pm5a* | 5 | 7 | 9 | 9 | 6 | 8 | 8 | 9 | 3 | 8 | 8 | 2 | 3 | 3 | 5 |
| TP114/2*Starke | *Pm6* | 6 | 8 | 8 | 8 | 8 | 8 | 8 | 9 | 9 | 9 | 9 | 8 | 9 | 8 | 9 |
| Disponent | *Pm8* | 2 | 9 | 8 | 8 | 7 | 9 | 7 | 9 | 8 | 7 | 6 | 7 | 8 | 8 | 8 |
| Wembley 14.31 | *Pm12* | 2 | 2 | 9 | 7 | 4 | 1 | 1 | 1 | 8 | 8 | 5 | 5 | 8 | 8 | 7 |
| Amigo | *Pm17* | 5 | 7 | 9 | 3 | 8 | 1 | 9 | 9 | 9 | 1 | 1 | 9 | 8 | 8 | 9 |

Table S2 Primer sequences of the two KASP markers for QTL *QPm.NOBAL-3A* developed in this study.

| Marker name | Forward primer 1 | Forward primer 2 | Common reverse primer |
| --- | --- | --- | --- |
| AX-94555538 | TCTGACAATCTTTGGGAATGCA | TCTGACAATCTTTGGGAATGCG | CAGCAGCTCGCACCTACATA |
| RFL_Contig1488_671 | CTCAGGGCAGGATCGACGT | CTCAGGGCAGGATCGACGC | TTCCGACTTCTTCCAGTCCAA |
